# Epigenetic Modulation of CD8^+^ T Cell Function in Lentivirus Infections: A Review

**DOI:** 10.3390/v10050227

**Published:** 2018-04-28

**Authors:** Mukta Nag, Kristina De Paris, Jonathan E. Fogle

**Affiliations:** 1 Department of Population Health and Pathobiology, College of Veterinary Medicine, North Carolina State University, 1060 William Moore Drive, Raleigh, NC 27607, USA; mnag@ncsu.edu; 2 Department of Microbiology and Immunology, University of North Carolina at Chapel Hill, Chapel Hill, NC 27599, USA; kristina_abel@med.unc.edu

**Keywords:** CD8^+^ T cells, T regulatory cells, CD8^+^ T cell dysfunction, epigenetics, lentiviral infections, feline immunodeficiency virus, simian immunodeficiency virus, human immunodeficiency virus

## Abstract

CD8^+^ T cells are critical for controlling viremia during human immunodeficiency virus (HIV) infection. These cells produce cytolytic factors and antiviral cytokines that eliminate virally- infected cells. During the chronic phase of HIV infection, CD8^+^ T cells progressively lose their proliferative capacity and antiviral functions. These dysfunctional cells are unable to clear the productively infected and reactivated cells, representing a roadblock in HIV cure. Therefore, mechanisms to understand CD8^+^ T cell dysfunction and strategies to boost CD8^+^ T cell function need to be investigated. Using the feline immunodeficiency virus (FIV) model for lentiviral persistence, we have demonstrated that CD8^+^ T cells exhibit epigenetic changes such as DNA demethylation during the course of infection as compared to uninfected cats. We have also demonstrated that lentivirus-activated CD4^+^CD25^+^ T regulatory cells induce forkhead box P3 (Foxp3) expression in virus-specific CD8^+^ T cell targets, which binds the interleukin (IL)-2, tumor necrosis factor (TNF)-α, and interferon (IFN)-γ promoters in these CD8^+^ T cells. Finally, we have reported that epigenetic modulation reduces Foxp3 binding to these promoter regions. This review compares and contrasts our current understanding of CD8^+^ T cell epigenetics and mechanisms of lymphocyte suppression during the course of lentiviral infection for two animal models, FIV and simian immunodeficiency virus (SIV).

## 1. Introduction

**Figure 1:**
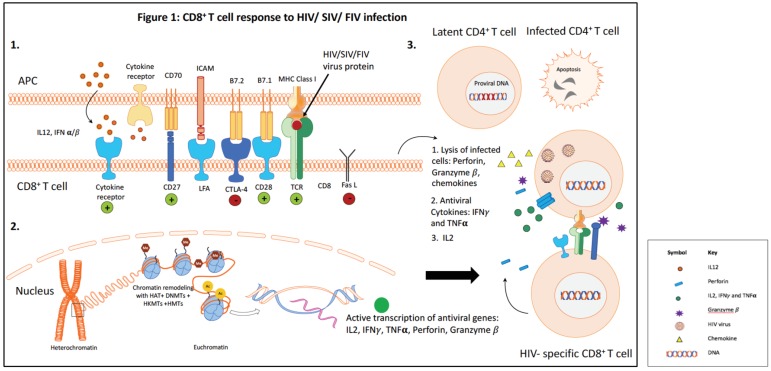
CD8^+^ T cell response to human immunodeficiency virus (HIV)/simian immunodeficiency virus (SIV)/feline immunodeficiency virus (FIV) infection. Part 1: Interaction of a professional antigen presenting cell (APC), e.g., dendritic cells, with a CD8^+^ T cell during lentivirus infection. The APCs present the viral antigens on the surface via major histocompatibility complex (MHC) Class I molecules for recognition by the T-cell receptor (TCR) on CD8^+^ T cells. Three signals are needed for CD8^+^ T cell activation. The first signal is provided by the engagement of the CD8^+^ TCR with MHC Class I on the APC cell surface presenting the viral peptide. The second signal is provided by the engagement of co-stimulatory molecules, here CD28, on the CD8^+^ T cells with CD80 (B7.1) on the APCs. Cytokines provide the third signal required for T cell proliferation. As the CD8^+^ T cells become activated, they also begin upregulating cytotoxic T lymphocyte-associated molecule (CTLA)-4. CTLA-4 binding with CD86 (B7.2) on APCs delivers an inhibitory signal to maintain immune homeostasis. Part 2: Upon CD8^+^ T cell activation, the chromatin within the nucleus “relaxes” into euchromatin conformation to allow the binding of various factors of the transcriptional machinery. Chromatin remodeling enzymes such as histone acetyltransferases (HAT), histone methyltransferases (HMTs), DNA methyltransferases (DNMTs) and histone lysine methyltransferases (HKMTs) alter the accessibility of chromatin at specific sites based on the signals provided to direct the specific response. During HIV infection, “relaxed” chromatin in gene promoter regions allows for the active transcription of antiviral genes such interleukin (IL)-2, interferon (IFN)-γ, tumor necrosis factor (TNF)-α, perforin and granzyme β. Part 3 illustrates the interaction of activated HIV-specific CD8^+^ T cells with productively HIV-infected CD4^+^ T cells. Upon recognition, the infected CD4^+^ T cells undergo lysis and apoptosis due to the action of perforin, granzyme β, chemokines, IFN-γ and TNF-α. IL-2 expressed by activated CD8^+^ T cells aids in T cell proliferation. Despite a robust HIV-specific CD8^+^ T cell response in the acute phase, latently infected CD4^+^ T cells escape immune recognition and may become reactivated later during the infection. Effective reactivation and elimination of these latently infected cells is one of the major obstacles to HIV cure.

CD8^+^ T lymphocytes (CTLs) play a critical role in the control of human immunodeficiency virus (HIV) infection [1,2]. The HIV-specific CD8^+^ T cell response peaks just after peak viremia during the acute phase of infection, and then reaches a lower level steady state in the chronic phase of infection [3]. Efficient control of HIV replication by HIV-specific CD8^+^ T cells is attributed to both the cytotoxic function and the polyfunctional cytokine response [4]. Despite the robust antiviral cytolytic CTL response in the acute phase, lentiviruses are able to escape immune recognition and persist in the host. Long-term viral persistence results in continued immune activation, and this in turn impairs the CD8^+^ T cell response. CD8^+^ T cells become progressively dysfunctional as the lentivirus infection proceeds. Dysfunctional CD8^+^ T cells exhibit reduced proliferative capacity and defective effector function, including decreased production of the antiviral cytokines interleukin (IL)-2, interferon (IFN)-γ and tumor necrosis factor (TNF)-α [5,6]. Figure 1 summarizes the CD8^+^ T cell response during HIV infection.

The introduction of combination antiretroviral therapy (cART) has transformed HIV/acquired immune deficiency syndrome (AIDS) from a fatal disease to a chronic disease with almost normal life expectancy. The new challenge in our fight against the HIV/AIDS epidemic is now finding a cure. The biggest obstacle in cure research is developing an effective strategy that can reactivate latently infected cells and at the same time eliminate the purged virus and virally infected cells [7,8,9]. CD8^+^ T cells are vital in the process of eliminating infected cells [10,11,12]. However, chronically activated CD8^+^ T cells that are functionally impaired are unable to clear the infection [13,14]. Therefore, there is a critical need to identify novel methods to boost and restore CD8^+^ T cell function.

To achieve this goal, we need to understand the molecular mechanisms leading to CD8^+^ T cell dysfunction. Exhausted CD8^+^ T cells can be phenotypically characterized by the expression of inhibitory molecules, such as programmed cell death (PD)-1, cytotoxic T lymphocyte-associated molecule (CTLA)-4, lymphocyte activation gene (LAG)-3, T-cell immunoglobulin and mucin-domain containing (TIM)-3, and T-cell immunoreceptor with IgG and ITIM domains (TIGIT), that interfere with effective T cell activation [15,16]. In HIV infection and the rhesus macaque model of simian immunodeficiency virus (SIV) infection, checkpoint inhibitors targeting these molecules can partially restore CD8^+^ T cell function [17,18,19]. These studies provide proof-of-concept for the feasibility of developing effective immune strategies to enhance CD8^+^ T cell function in vivo.

**Figure 2:**
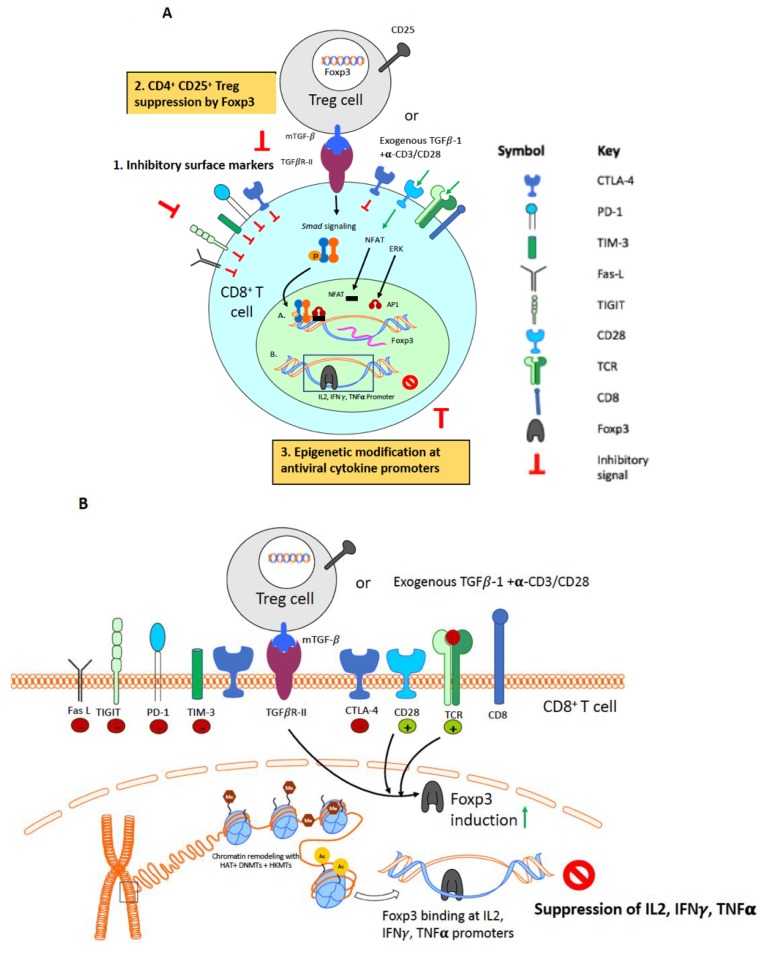
Persistent antigenic stimulation results in CD8^+^ T cell dysfunction during chronic HIV infection. Figure 2A illustrates three mechanisms of CD8^+^ T cell dysfunction during chronic HIV infection. Part 1: Upregulation of inhibitory surface markers such as programmed cell death (PD)-1, cytotoxic T lymphocyte-associated molecule (CTLA)-4, T-cell immunoglobulin and mucin-domain containing (TIM)-3, T-cell immunoreceptor with IgG and ITIM domains (TIGIT) and Fas-L that inhibit effective CD8^+^ T cell activation. Part 2: Treg-mediated suppression. Treg-mediated suppression has been observed in HIV-patients, SIV-infected macaques and in FIV-infected cats. Our lab, using the FIV model of HIV infection, has demonstrated that lentivirus-activated Treg cells upregulate membrane-bound tumor growth factor (TGF)-β which ligates the TGF-βRII on activated CD8^+^ T cells. This ligation leads to downstream phosphorylation of the Smad complex, resulting in its nuclear translocation. In addition, CD8^+^ T cell activation and signaling via the TCR and CD28 co-stimulatory molecule promotes the co-operation of nuclear factor of activated T-cells (NFAT): activator protein (AP)-1 within the nucleus, an interaction required for gene activation in T cells. Smad complexes coupled with NFAT:AP-1 binding at the forkhead box P3 (Foxp3) promoter results in the induction of the repressive transcription factor *Foxp3*. Foxp3 induction in CD8^+^ T cells can also be induced by exogenous addition of TGF-β1 if simultaneous TCR activation via anti-CD3/anti-CD28 occurs. Part 3: Epigenetic modulation of antiviral cytokine genes and their regulatory regions. As a result of CD8^+^ T cell activation (see Figure 1, Part 2), antiviral cytokine promoters are “relaxed”. This open conformation allows repressive transcription factors such as Foxp3 to bind and thereby suppress the transcription of IL-2, IFN-γ and TNF-α as shown in Figure 2B.

In this review, we will discuss additional mechanisms that may be exploited to enhance CD8^+^ T cell function. Specifically, we will focus on the role of regulatory CD4^+^ T cells (Treg cells) and epigenetic modifications in the regulation of CD8^+^ T cell function. Figure 2A illustrates some of the main mechanisms leading to the generation of dysfunctional CD8^+^ T cells.

## 2. Animal Models of HIV Infection to Study CD8^+^ T Cell Dysfunction

Animal models provide a useful tool to study HIV infection, its pathogenesis and cure strategies. The three most commonly used animal models in HIV research are the humanized mouse model, the FIV model, and the SIV model in non-human primates (NHP). Humanized mice represent a relatively simple, rapid and small-sized animal model used in HIV research. These mice are immunocompromised animals that are reconstituted, in part, with human immune cells. Humanized mice, specifically severe combined immunodeficient (SCID)-hu mice have been instrumental to documenting the existence of viral latency and understanding latently infected cells [20,21]. Initial investigations using latency reversing agents (LRAs) demonstrating the activation of HIV-1 and one of the earliest “shock and kill” experiments was shown in SCID-hu mice [21]. The humanized-mouse model and its utilization in HIV research have been reviewed in Policicchio et al. [22]. In this review, we will be discussing the FIV and SIV models of HIV infection.

The FIV infection model represents the only animal model of a naturally occurring immunodeficiency infection caused by a lentivirus. This outbred model is representative of HIV disease progression in humans [23,24,25]. FIV, akin to HIV, is transmitted by blood transfer, mucosal contact and vertically via prenatal and postnatal routes [26,27]. During FIV infection, there is a short acute phase lasting 4–8 weeks, followed by a protracted asymptomatic phase of chronic infection spanning several months to years. The type of infecting FIV strain and the genotype of the cat significantly influence the rate of disease progression. Infection eventually leads to the development of immunodeficiency symptoms consistent with the disease progression in HIV-infected patients (reviewed in Elder et al. [24]). FIV-infected cats exhibit high viremia during the acute phase, and, as the disease progresses, the drop in CD4^+^ T cells causes a sharp increase in viral RNA levels ultimately leading to immune dysfunction [24]. The compromised immune system increases the risk of muscle wasting, opportunistic infections and neoplasia in cats, analogous to humans [28]. This phase is defined as feline acquired immunodeficiency syndrome (FAIDS) [23]. Certain strains of FIV are neurovirulent and have degenerative effects on the central nervous system similar to the neurological impairment seen during HIV-1 infection [29,30,31]. A commercially available prophylactic vaccine against FIV (Fel-O-Vax FIV by Boehringer Ingelheim) was released in the U.S. in 2002 and informed the identification of conserved vaccine epitopes for HIV vaccine development [32]. The FIV model has been extensively used for the development and efficacy studies of antiretroviral drugs for HIV treatment. Early studies determining the in vivo efficacy of acyclic nucleoside phosphonate analogues in the FIV model were instrumental in the development of tenofovir—one of the most commonly used drugs in the ART regime [33,34]. FIV is sensitive to nucleoside-analogue reverse-transcriptase inhibitors (NRTIs) and HIV-1 integrase inhibitors, enabling efficacy studies of these classes of drugs [35,36,37,38,39]. Transgenic cats, genetically engineered to express HIV-specific restriction factors such as TRIMCyp, a fusion protein derived from the tripartite motif-encoding protein (*TRIM*)-*5* gene have improved the translational relevance of the FIV model to study HIV infection and pathogenesis [40]. FIV has also been studied as a model for lentivirus latency (reviewed in McDonnel et al. [25]). The FIV promoter in latent in vivo CD4^+^ T cells exhibits de-acetylated histones suggesting a repressive transcriptional state consistent with the findings in highly active antiretroviral therapy (HAART) treated HIV patients [41,42]. Therefore, the FIV model was applied to study the effects of the LRA suberoylanilide hydroxamic acid (SAHA) in reactivating latent viral reservoirs [43]. In summary, the FIV model has enabled the testing of multiple treatment and lentivirus control strategies that are not feasible to perform in HIV-infected patients. 

This model has, however, some limitations. There are differences between the FIV and HIV-1 viral genome. FIV lacks the viral protein R (*vpr*), viral protein U (*vpu*) and negative regulatory factor (*nef*) equivalent genes, and does not encode the transactivator of transcription (Tat) protein or the sequence for the transactivation response element (Tar) that are present in the HIV-1 genome. Instead, FIV expresses OrfA, an accessory protein with multiple functions enabling enhanced viral transcription, viral particle formation, viral release from infected cells and viral infectivity, which is not encoded by the HIV genome [24,44]. FIV OrfA localizes to the nucleus and its transient expression induces G2 cell cycle arrest suggesting its similarity with HIV Vpr protein [45].

FIV uses CD134 as the main binding receptor instead of CD4, enabling the virus to infect B cells and CD8^+^ T cells along with CD4^+^ T cells and macrophages, whereas CD4^+^ T cells and macrophages serve as the main target cells in HIV infection [46,47]. Due to the difference in FIV and HIV targets, the reservoir dynamics differ between the two infections (reviewed in Policicchio et al. [22]). However, in a recent study CD4^+^ and CD21^+^ leukocytes were identified as cellular reservoirs for FIV replication which is similar to the subsets identified in HIV infection wherein CD4^+^ T cells serve as cellular reservoirs and CD21^+^ B cells serve as extracellular HIV reservoirs [48,49]. The same study identified FIV reservoirs in tissues such as the spleen, the intestine and mesenteric lymph node with B cell follicles as the foci of viral replication in chronically infected cats exhibiting viral latency [50]. This demonstrates that the FIV model offers the advantage of performing longitudinal infection studies which can be beneficial in understanding tissue viral reservoir dynamics [50]. Despite the great promise in expanding our understanding of HIV infection and treatment by utilizing this feline model, more translationally relevant models are needed to more accurately reflect HIV pathogenesis.

The most widely used in vivo animal model for HIV is the NHP model of SIV infection. Non-human primates and humans share several important aspects of genomic structure, physiology, and complexity of the immune system [51]. These shared features enable the widespread use of the NHP SIV model to study HIV pathogenesis, prevention, treatment and cure strategies. There is a wide range of NHP models that are used for HIV studies, each with its respective limitations. African Green monkeys and apes are the natural hosts for SIV, but generally the infection does not manifest into a disease-state. Asian macaques, which are not natural hosts for SIV, can be infected with certain strains of SIV leading to high viremia, CD4^+^ T cell depletion and an immunocompromised state characterized by tumors and opportunistic infections. The main features of SIV infection in natural and non-natural hosts have been contrasted and reviewed in Chahroudi et al. and Evans et al. [52,53]. Rhesus macaques of Indian origin and pig-tailed macaques are the most commonly used NHP model for AIDS research [53]. All modes of HIV transmission can be recapitulated in these NHP models. Many antiretroviral (ARV) drugs, such as tenofovir, zidovudine, nevirapine and emtricitabine, which are now used to treat HIV-infected patients, were first tested for efficacy in the macaque models [54,55,56]. Due to the similarities in metabolism and physiology, macaque models have been invaluable in toxicity and dose testing studies of ART drugs. These drugs have informed pre-exposure prophylaxis and post-exposure prophylaxis treatment regimes in humans. Macaque studies also established that early treatment with ART reduces HIV-viremia, delays disease progression, enhances antiviral responses and lowers the risk of transmission [3,55]. Analogous to ARV testing in the NHP model, NHP models have been instrumental in the testing of first-generation (mostly surfactants and poly anionic compounds) and second-generation (antiretrovirals) microbicides that confer local mucosal antiviral protection against HIV infection (reviewed by Van Rompay [54]). The high translational impact of NHP research is best represented by the success of the CAPRISA trial in which one percent tenofovir gel demonstrated partial efficacy in protecting women against HIV infection [57]. 

The NHP model has been widely used to test HIV vaccine strategies. In fact, the outcomes of the three major phase III HIV vaccine trials, i.e., Vax003, Vax004 and RV144 trials, were predictable by prior NHP studies [58,59,60]. A new HIV phase 2b vaccine trial was launched in November of 2017 that will test an adenovirus 26 vector (Ad26) expressing four mosaic HIV proteins (Ad26.Mos4HIV) in combination with clade C gp140 protein boosting. This vaccine had shown protective efficacy against simian–human immunodeficiency virus (SHIV) infection in adult rhesus macaques [61].

In recent years, NHP models have been extensively used to study the formation and maintenance of the viral reservoir, and to evaluate cure strategies. SIV integrates into the host genome at preferred integration sites [62,63]. Similar to HIV, SIV undergoes histone de-acetylation in long terminal repeats (LTRs) and these changes contribute to latency [64,65]. CTLs are unable to clear latently SIV-infected cells [66]. As the distribution patterns of HIV and SIV DNA and RNA in peripheral blood, lymph nodes and mucosal tissues are also very similar, NHP models likely accurately reflect reservoir dynamics of HIV-infected humans [67,68,69]. A potential limitation of the SIV infection model is the use and efficacy of specific ARVs in this model. However, with the development of novel chimeric SHIVs such as reverse transcriptase-SHIV (RT-SHIV) and SHIV clones with HIV-1 Env proteins, clinically relevant NHP models can be established [70,71,72]. However, the NHP model, despite its widespread application in HIV cure research, remains greatly unexplored for epigenetic studies defining lentivirus infections.

## 3. CD8^+^ T Cell Suppression by CD4^+^CD25^+^ T Regulatory Cells

Treg cells are immunomodulatory cells that control exaggerated immune activation upon infection, thereby mitigating the extent of tissue damage. These cells express high levels of the repressive transcription factor Foxp3, express the high affinity IL-2 receptor CD25 and low levels of the IL-7 receptor (CD127) [73,74,75,76]. Two types of Treg cells have been described. Natural Treg cells (nTreg) are derived from the differentiation of CD4^+^CD8^+^ precursors into Foxp3^+^ CD4^+^CD25^+^ T cells in the thymus. Whereas CD4^+^ T cells that are induced and mature into Foxp3^+^CD4^+^CD25^+^ T cells in the periphery are called induced Treg cells (iTreg) [77,78,79]. iTreg cells are comparable to thymus-derived nTreg cells in suppressor function and express similar transcription factors and surface markers [78,80,81]. Foxp3^+^ Treg cells can also be induced in vitro by T-cell receptor (TCR)-mediated activation of naïve CD4^+^CD25^−^ T cells in the presence of TGF-β and IL-2 [82,83]. They regulate the immune response by inhibiting T cell proliferation by competing for the growth factor IL-2, by CTLA-4 binding to dendritic cells (DCs), through the secretion of inhibitory cytokines such as IL-10 and TGF-β, and utilizing cytolytic molecules such as granzyme B and perforin [84]. While Treg cells play a critical role in maintaining self-tolerance, they also suppress antigen-specific immune responses that enable the pathogen to persist [85,86].

In HIV infection, Treg cells can have positive or detrimental effects depending on the stage of disease, viral load, tissue and cell type. Upon infection, Foxp3 has been reported to inhibit HIV transcription through inhibition of nuclear factor of activated T-cells (NFAT) and nuclear factor (NF)-κB activation in vitro [87], whereas other studies suggest that Foxp3 enhances HIV gene expression via the *NF–κB* signaling pathway [88]. Chronic HIV infection leads to an expansion of Treg cells in peripheral blood and lymphoid tissues; preferentially in regions with active HIV replication, such as lymphoid and mucosal tissues [89,90]. This expansion has been attributed to multiple reasons, including persistent immune activation, increased survival of Treg cells, and increased generation of CD4^+^CD25^+^ Foxp3^+^ Treg cells in the thymus of HIV-infected patients [91]. An increased frequency of Treg cells correlates with lower CD8^+^ T cell activation in HIV-1 infection [17]. An early induction of Foxp3^+^Treg cells in the blood and an early accumulation of Treg cells in mucosal tissues and peripheral lymph nodes is demonstrated in the nonpathogenic model of African green monkeys and pathogenic model of rhesus macaques when infected with SIV respectively [92,93]. There is a rapid depletion of Treg cells in the pathogenic model of pigtailed macaques infected with SIV [94,95]. In the FIV model, Treg cells are phenotypically and functionally activated during the acute phase and remain activated through the chronic phase of infection [96]. These combined findings indicate that Treg frequency, longevity and accumulation dynamics are influenced by multiple factors.

Treg cells are susceptible to HIV infection because they also express the HIV co-receptors C-C motif chemokine receptor 5 (CCR5) and C-X-C chemokine receptor 4 (CXCR4). Indeed, both human and animal studies demonstrate that Treg cells support HIV-1, FIV and SIV replication in vitro and in vivo [97,98,99]. A very low percentage of peripheral Treg cells are infected by HIV-1 in vivo (<0.7% peripheral Treg cells) [100]. SIV infected Foxp3^+^ T cells are found in multiple tissues, including mucosal tissues such as gut-associated lymphoid tissue [92,97,101]. FIV+ cats harbor productively infected Treg cells that are phenotypically and functionally activated [102]. Treg cells in HIV-infected humanized mice also support high levels of HIV-1 which are depleted upon infection with HIV-1 [103].

This review will focus on the interaction of Treg cells with CD8^+^ T cells during lentivirus infections and the resulting suppression of antiviral CD8^+^ T cell function. There are conflicting reports on the role and the functional capacity of Treg cells in HIV infection [104]. For example, Treg cells in HIV-1 infected patients support HIV infection which results in the downregulation of Foxp3 and impairment of their suppressive capacity when assessing individual cells [105,106]. In contrast to these studies, in vitro and other in vivo studies suggest that bulk Treg cells retain, or in some cases enhance their immunosuppressive function during the course of HIV-1 and FIV infection [88,100,102,106,107].

Treg cells isolated from HIV-infected patients suppress cytolytic function of HIV-specific CD8^+^ T cells [90]. Similarly, increased Treg frequencies during acute SIV infection correlate with suppressed SIV-specific CD8^+^ T cell responses [97]. The effector T cells also enhance their sensitivity towards Treg-mediated suppression in HIV-1 infected patients [108]. Enhanced HIV/SIV/FIV-specific T cell responses upon ex vivo depletion of Treg cells from peripheral blood mononuclear cells (PBMCs) or lymphoid cell suspensions support the immunosuppressive role of Treg cells [109,110]. CD8^+^ T cells from HIV-infected patients with the protective major histocompatibility complex (MHC) human leukocyte antigen (HLA) B*27 and HLA B*57 alleles evade Treg suppression, further suggesting that the mechanism of Treg suppression of CD8^+^ T cell functions is important for disease progression [111]. Nikolova et al. recently reported that Treg cells signal via PD-1/PD-L1 pathway to suppress HIV-specific CD8^+^ T cells contributing to CD8^+^ T cell dysfunction during the chronic infection phase [112]. Our group has previously reported that, in the FIV model, lentivirus activated CD4^+^CD25^+^ Treg cells signal via membrane bound TGF-β/Smad signaling to induce Foxp3 in CD8^+^ T cell targets [113,114]. We have also demonstrated that Foxp3 mediates antiviral cytokine suppression by directly binding to IL-2, TNF-α and IFN-γ promoter region in the acute and chronic phase of FIV infection [107,115,116]. Figure 2B summarizes our findings demonstrating Treg-mediated CD8^+^ T cell suppression in the FIV model. Based on our findings, this review will focus on the immunosuppressive function of Treg cells, but we acknowledge that Treg cells can also have beneficial effects on HIV infection and control, as reported by other investigators (reviewed in Moreno et al. [110]).

## 4. Epigenetic Mechanisms Regulating Gene Transcription: A General Overview

Epigenetic modifications are alterations in DNA and nucleosomes that occur in response to changes in the cellular environment. These changes are independent of changes in the primary DNA sequence and are reversible. Epigenetic modifications provide a second layer of transcriptional control by altering chromatin accessibility for the transcriptional machinery to bind and enable gene expression. Following cellular activation, the epigenetic patterns within the gene and its regulatory regions change to enable transcription factors to bind and initiate transcription of required genes. 

There are different types of epigenetic modifications, and we will briefly review them here. Histone modifications and changes in DNA methylation are two of the better understood epigenetic mechanisms that regulate gene transcription. Table 1 summarizes the most well characterized histone modifications and their effects upon gene expression.

DNA wraps around histones forming the main structural unit of nucleosomes. The N-terminus of the histone is susceptible to a variety of post-translational modifications such as acetylation, methylation, phosphorylation, ubiquitination and sumoylation that alter the affinity of nucleosome for DNA [117]. Histone methyltransferases (HMTs) and demethylases work to modify the methylation status of histone tails, thereby promoting an active or repressive chromatin conformation. The extent of histone lysine and serine methylation determines the activation or repression of specific genes (see Table 1). Acetylation of histone lysine residues is generally associated with a non-repressed state. Histone acetyltransferases (HATs) and histone deacetylases (HDACs) mediate histone acetylation and deacetylation respectively, changing the affinity of the nucleosome for DNA.

Promoters of “active” genes have acetylated histones and possess other activating histone marks such as H3K4me3 (histone H3 trimethylated on lysine 4) [123] (Table 1). Silencing of genes is a complex process which involves a wide range of proteins. Methyl-cytosine binding proteins (MBP) recruit deacetylases resulting in de-acetylated histones: H3 and H4. Other repressive modifications such as H3K9me3 (histone H3 trimethylated on lysine 9) and H3K27me3 (histone H3 trimethylated on lysine 27) are also found in silenced genes/promoters (Table 1) [124].

In addition to histones, direct methylation of DNA represents another epigenetic program that can regulate chromatin accessibility and hence gene transcription. In mammals, DNA methylation occurs at the C5-position[5-mc) of cytosine residues in the context of CpG dinucleotides where a guanine follows a cytosine nucleotide in a DNA sequence [125,126,127]. The frequency of CpG dinucleotides is 10 times greater than average in CpG islands (CGI) [128]. Generally, CpGs outside the CGIs are mostly methylated, whereas the CpGs within these islands are prone to de-methylation, making them important for gene regulation [124,128,129]. Methylation of CpG residues is usually associated with a “closed” chromatin (heterochromatin) structure resulting in gene suppression [130,131]. CpG methylation patterns are maintained or added de novo by DNA methyltransferases 1, 3a and 3b respectively (DNMT 1, 3a and 3b). Active promoters are usually un-methylated and result in an “open” chromatin conformation (euchromatin).

Both histone modifications and DNA methylation alter chromatin accessibility. These changes enable the transcriptional machinery to bind to promoters or other regulatory regions of genes, thereby controlling their level of expression. A single promoter can have a combination of these modifications simultaneously, that instruct a gene for active transcription or suppression. As the chromatin “opens” for transcription, it also allows repressive transcription factors to bind to gene regulatory and/or promoter regions resulting in gene suppression. Therefore, epigenetic modulation of gene expression is a complex process that provides a secondary level of gene regulation.

## 5. Epigenetics in FIV and SIV

Epigenetic changes in the FIV and SIV proviruses have been extensively studied to understand HIV latency which have enabled the testing of ARVs in both the disease models. The FIV and SIV LTR regions of promoters have de-acetylated and methylated histones similar to HIV infection resulting in latency [42,64,65]. Unlike during HIV infection, the FIV proviral promoter is not hypermethylated in latently infected CD4^+^ T cells and monocytes isolated from peripheral blood of FIV-infected cats [132]. During the asymptomatic phase of SIV infection, H4 is de-acetylated in the brain whereas in the acute phase, high levels of acetylated H4 are detected [64]. As described in previous sections, HDAC and HMT inhibitors such as SAHA and valproic acid have been tested in these two animal models successfully reactivating latent FIV/SIV in vitro and ex vivo [25,65]. Studies exploring the role of epigenetics in immune cells responding to lentivirus infections is limited. Our group largely studies the epigenetic events in CD4^+^ and CD8^+^ T effector cells due to the interaction with Treg cells during FIV infection. We have demonstrated that during FIV infection, CD8^+^ T cells directly interact with lentivirus activated Treg cells via a membrane bound TGF-β mechanism, inducing Foxp3 in these cells [113,114]. We have shown that Foxp3 binds to the IL-2, IFN-γ and TNF-α promoters in CD8^+^ T cells isolated from FIV-infected cats and that this binding is epigenetically modulated, via DNA de-methylation and histone acetylation ([115,116]; manuscript in preparation). We have also reported the microRNA 10a is upregulated in Foxp3^+^ Treg cells isolated from FIV-infected cats likely stabilizing Foxp3 expression in Treg cells ex vivo [133]. Collectively, our data suggest the role of epigenetics in explaining, in part, the mechanism of Treg-mediated suppression of CD8^+^ T cells during chronic lentivirus infections using the FIV model. Although the SIV model has not been utilized to characterize the epigenetic landscape in response to SIV infection, recent reports highlight the importance of studying this model to further elucidate the molecular events driving CD8^+^ T cell dysfunction. A recent study identified the differentially methylated transcription factors and microRNAs in inter and intra-species analysis between humans and rhesus macaques [134]. MicroRNA mml-miR-338-5p is differentially methylated and modulated by SIV infection in the brain after treatment with cannabinoids, frequently prescribed to people living with HIV/AIDS (PLWHA) [135]. Another recent report discussed the potential role of H3K27 methylation by enhancer of zeste homolog 2 (EZH2) in the differentiation and maturation of B and T follicular helper (Tfh) cells in the germinal centers (GC) during SIV infection [136]. These reports clearly suggest that epigenetic changes drive the cellular responses during SIV infection. Therefore, there is a need to further elucidate the epigenetic events involved in cell-mediated immune responses to lentivirus infections, specifically CD8^+^ T cells.

## 6. Epigenetics in HIV

There are multiple reports describing epigenetic modifications during chronic viral infections such as HIV, hepatitis B virus (HBV), hepatitis C virus (HCV), human papillomavirus (HPV) and Epstein–Barr virus (EBV) infections in humans [137,138,139,140,141]. For example, HBV and HCV induce DNA methylation in mice with humanized livers and the HPV viral genome is subjected to DNA methylation-mediated control during infection [141,142]. The epigenetic alterations in these chronic viral infections and the use of epigenetic therapy for their treatment has been reviewed in Moos et al. [143].

Epigenetic modifications in HIV infection been extensively studied in the context of HIV latency. HIV proviral latency is maintained by histone deacetylases that inhibit transcription from the HIV LTR [144]. Deacetylation and methylation of histones during HIV-1 infection interfere with the binding of DNA polymerase and restrict the production of active HIV-1 transcripts from proviral DNA. The 5′ long terminal repeat (5′ LTR) region of HIV DNA is highly susceptible to inhibitory epigenetic modification [41]. Silencing epigenetic modifications of other viral proteins are a major contributor to viral latency. The genes of several HIV accessory proteins, such as *Nef*, *Vpr* and *Tat*, are hyper-methylated and thereby promote viral latency [145,146]. As these epigenetic modifications are reversible, efforts have been made to identify epigenetic modulators that can reactivate these silenced viral genes, and thereby reactivate latently infected cells. The “Shock and Kill” strategy aims to reverse latency by manipulating histone post-translational modifications to enable virus replication; once virus is activated and released, multiple different strategies are being pursued to eliminate virally infected cells or free virus. The latter include suppression of virus replication by interfering with the virus life cycle through antiretroviral drugs or the boosting of antiviral immune responses [11]. HDAC inhibitors such as valproic acid, SAHA (vorinostat) and panobinostat have been reported to increase HIV-1 transcription from proviral DNA in latently infected cells in vitro and in clinical trials [147,148,149]. Similarly, treatment with the histone lysine methyl transferase inhibitor, HKMT G9a antagonist BIX01294, has resulted in increased HIV RNA levels, and combination therapy approaches utilizing different HDAC inhibitors and HKMT inhibitors are being explored [150,151]. Other studies have tested the manipulation of epigenetic moieties at the DNA level by using modulators such as 5-aza-2′- deoxycytidine (aza-CdR) [152]. Apart from epigenetic modifiers, several small molecules, such as JQ1 and protein kinase C (PKC) agonists can reactivate the latent reservoirs as well.

An important question that needs to be pursued in parallel is whether the patient’s immune response will be robust enough to clear the infection upon successful purging of the reservoir. As described above, virus-specific CD8^+^ T cells exhibit progressive dysfunction during the course of infection due to a number of factors including persistent immune activation, Treg-mediated suppression, and lentivirus induced epigenetic modifications. Thus, to successfully cure HIV infection, we need to address this problem from multiple angles. Along with latency reactivation, it is important to identify strategies that can boost CD8^+^ T cell function to eliminate reactivated cells.

## 7. Epigenetic Modulation of Immune Cells as a Result of HIV Infection

In order to develop strategies to boost CD8^+^ T cell function, we need a better understanding of the epigenetic changes in immune cells infected with and responding to HIV infection, including CD8^+^ T cells, and this will be the focus here. Recent studies indicate that epigenetic modifications are induced very early after infection [153]. In vitro, epigenetic modifications in PBMC or primary CD4^+^ T cells can be detected as early as 36 hours after HIV-1 infection [153]. Enrichment of H3K9me3 and H3K27me3 marks result in transcriptional repression, including the downregulation of important antiviral cytokine genes, such as IL-2 and IFN-γ [153]. Similarly, Mikovitz et al. reported that increased methylation of the IFN-γ promoter along with increased DNMT expression results in the suppression of IFN-γ in HIV-infected CD4^+^ T cell in vitro [154]. In another study, Hosoya et al. demonstrated that DNA methylation during chronic HIV infection regulates loss of IL-2 in senescent CD4^+^ T cells [155]. They also reported that the CD28 co-stimulatory signaling pathway plays an important role in de-methylation/re-methylation of the *Il2* gene [155]. Both IL-2 and IFN-γ are necessary to support CTL and natural killer (NK) cell activity and thus, the suppression of these cytokines in CD4^+^ T cells might negatively impact virus control during HIV infection [156].

In addition, CD8^+^ T cells themselves are epigenetically modified during HIV infection [157,158]. For example, the expression of the inhibitory PD-1 molecule is regulated by methylation of the PD-1 promoter. CD8^+^ T cells from HIV-infected subjects classified as long term non-progressors (LTNP) or patients with fully suppressed plasma viremia by ART have an unmethylated PD-1 promoter [158]. Zhang et al. reported that the inability of exhausted CD8^+^ T cells to produce IFN-γ and IL-2 positively correlates with low levels of diAcH3 in the regulatory regions of the *Ifng* and *Il2* genes. They further demonstrated that exhausted CD8^+^ T cells have overall lower levels of histone acetylation [159]. These data imply that CD8^+^ T cell function is epigenetically controlled during HIV infection. Therefore, reversing repressive epigenetic signatures using epigenetic modulators could potentially recover CD8^+^ T cell function.

Our research group has studied the molecular events responsible for CD8^+^ T cell dysfunction in the FIV model. Data by our lab and others suggest that FIV provirus and infected CD4^+^ T cells are epigenetically modulated during infection and that epigenetic modulators can be used to reverse these events [25,115]. We have further demonstrated that dysfunctional CD8^+^ T cells, that evolve during the chronic phase of FIV infection, are epigenetically modulated [115]. Specifically, lentivirus activated CD4^+^CD25^+^ Treg cells induce Foxp3 expression in CD8^+^ T cells, and Foxp3 mediates antiviral cytokine suppression by directly binding to the IL-2, TNF-α and IFN-γ promoter regions [107,115,116]. We have also demonstrated that blocking DNA de-methylation and histone acetylation reduces Foxp3 binding to the IL-2 promoter in the FIV model ([115], manuscript in preparation). These data reveal a novel Foxp3-mediated mechanism contributing to CD8^+^ T cell dysfunction in lentivirus infections.

In addition, Treg cells themselves can be epigenetically modulated by HIV-1 infection [105]. HIV-1 infection results in downregulation of Foxp3 expression in Treg cells followed by loss of suppressive activity and alterations in cytokine expression profile. The same group found the CpG sites in the Foxp3 locus to be hypermethylated due to increased expression of DNMT3b [105]. Another report, however, showed increased frequency of Treg cells in the gut mucosa of HIV-1 infected patients due to decreased methylation of Foxp3 promoters in the gut associated T cells, causing the induction of iTreg cells [160]. They also reported that the Foxp3 promoter was significantly de-methylated possibly due to the downregulation of key DNMT enzymes in HIV patients when compared to uninfected control subjects [160]. These conflicting results regarding the methylation status of the Foxp3 promoter after HIV-1 infection indicate the lack of literature describing the epigenetic changes induced by lentivirus infections in Treg cells which necessitates further studies.

## 8. Current Strategies to Boost CD8^+^ T Cell Function

Several immune strategies have been tested to improve CD8^+^ T cell function during chronic HIV infection. Exhausted CD8^+^ T cells upregulate inhibitory receptors such as PD-1, CTLA-4, TIM-3, CD160, 2B4 and LAG-3, and in vitro and in vivo blockade of these inhibitory receptors has shown promising results in enhancing the cytotoxic function of CD8^+^ T cells [161,162]. Toll-like receptor 2 (TLR 2) agonists and agonistic antibodies against 4-1BB or CD40 have also yielded positive results in reversing CD8^+^ T cell exhaustion in vivo [163]. Additionally, recombinant cytokines alone or in combination with checkpoint inhibitors can increase CD8^+^ T cell function. For example, due to the success of recombinant IL-15 in enhancing CD8^+^ T cell function in animal models, the superagonist IL-15 ALT-803 is being tested in a clinical trial with ART-treated HIV patients [164,165].

In the past, adoptive transfer studies using functionally competent CD8^+^ T cells that were expanded ex vivo were not very successful, in part due to the absence of standardized protocols for ex vivo expansion of T cells and for a lack of knowledge on how to avoid non-specific immune activation [166,167]. However, with the recent advancements and success of the chimeric antigen receptor (CAR) technology in cancer therapy, CAR-T cells hold great promise in HIV-treatment. Recent reports described that T cells engineered to express anti-HIV CAR (HIV-CAR) can specifically target HIV-infected T cells ex vivo [168]. Epigenetic modulations using HDACi have been explored for their ability to boost CD8^+^ T cell function. Agarwal et al. demonstrated that the cytolytic response of stimulated CD8^+^ T cells can be increased by increasing the expression of IFN-γ, MIP-1α and MIP-1β using HDACi [169]. Similarly, in vitro treatment of exhausted CD8^+^ T cells with HDAC inhibitors could restore diAcH3 levels, improving CD8^+^ T cell function, and adoptive transfer of these cells resulted in long term persistence of these CD8^+^ T cells and development into functional memory cells in mice [159]. These studies support the rationale to further explore epigenetic modulators to reverse detrimental HIV-induced modifications and enhance antiviral host immunity.

## 9. Conclusions

**Figure 3:**
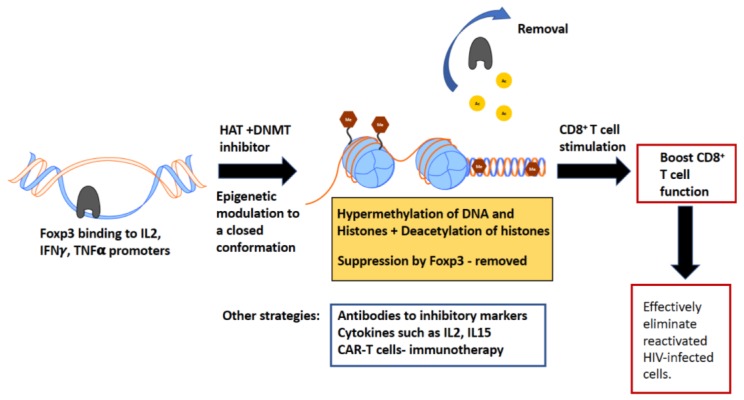
Proposed mechanism of epigenetic modulation to boost antiviral function of CD8^+^ T cells. In the FIV model, Treg-induced Foxp3 suppresses CD8^+^ T cell function by directly binding to the of IL-2, IFN-γ and TNF-α promoter regions. We have demonstrated that by blocking DNA de-methylation and histone acetylation using DNMT and HAT inhibitors respectively, Foxp3 binding to the IL-2 promoter is reduced. These data suggest that epigenetic modulations of dysfunctional CD8^+^ T cells can alleviate Foxp3-mediated suppression. We propose that these rescued CD8^+^ T cells, when stimulated, can restore their antiviral function and effectively eliminate reactivated virally infected cells. Other strategies to boost CD8^+^ T cell function include checkpoint inhibitors, immunotherapy with HIV-CAR T cells, and the use of stimulatory cytokines such as IL-15 and IL-2.

CD8^+^ T cell function is essential to fight chronic lentivirus infection and to eliminate reactivated latently infected cells. However, often these CD8^+^ T cells have impaired proliferation and functional capacity. We need to identify novel methods to boost these dysfunctional CD8^+^ T cells to improve current HIV cure strategies. Epigenetic manipulation of dysfunctional CD8^+^ T cells holds great promise to solve this problem. Figure 3 illustrates the mechanism of boosting CD8^+^ T cell function by epigenetically modulating dysfunctional cells.

In the FIV model, we have reported that Treg cells suppress CD8^+^ T cell antiviral function by inducing stable Foxp3 in CD8^+^ T cells, with Foxp3 directly binding to the promoters of IL-2, TNF-α and IFN-γ in FIV-specific CD8^+^ T cells [108,109]. We demonstrated that Foxp3 binding at these cytokine promoters is epigenetically modulated ([108]; manuscript in preparation), and by blocking DNA de-methylation and histone acetylation we could reduce Foxp3 binding to the IL-2 promoter region ([108]; manuscript in preparation). These data suggest that therapeutic interventions targeted at specific epigenetic modifications in dysfunctional CD8^+^ T cells can reverse Foxp3-mediated suppression. Currently, we are attempting to validate these findings in the rhesus macaque model of SIV infection. We are aiming to identify differences in the epigenetic signatures of SIV-infected and -uninfected macaques and whether epigenetic changes differ in distinct anatomic locations, such as mucosal tissues, secondary lymphoid tissues and peripheral blood. As epigenetic remodeling events can alter gene expression, we are specifically focusing on identifying epigenetic signatures associated with CD8^+^ T cell dysregulation. Utilizing the FIV and SIV animal models, our ultimate goal is to identify epigenetic targets that can be modulated for therapeutic use in HIV cure.

Several open questions remain: How early do these modifications appear during the course of infection? How informative will studies in animal models be? Are the epigenetic modifications across FIV, SIV and HIV conserved? Are there any differences in epigenetic signatures across CD8^+^ T cells with distinct differentiation states or in specific tissues? Are there differences in epigenetic signatures across different age groups, in HIV-infected people of different gender, or people in different geographic locations? How do virus-specific and/or host-specific factors influence epigenetic changes? Many other confounding factors, such as co-infection, are likely to alter the epigenetic landscape, too. Our understanding of epigenetic changes in immune cells important in viral control, i.e., CD8^+^ T cells, is very limited. Specifically, the knowledge of epigenetic signatures that are unique to CD8^+^ T cells remains relatively unknown. Therefore, we need to identify differences in the epigenetic landscape of CD8^+^ T cells in acute and chronic lentiviral infection. Only then can we identify specific epigenetic targets that can be modulated to improve the cytokine production and cytolytic function of dysfunctional CD8^+^ T cells during HIV infection.

## Figures and Tables

**Table 1:** Summary of epigenetic histone modifications and their effect on gene expression [118,119,120,121,122].

Histone/Position/Modification	Location	Effect	Enzyme
H3K4me2		Gene activation	Set1, MLL, Set7/9, SMYD3, LSD1, JAR1D1A
H3K4me3	5′ End of transcriptionally active genes	Gene activation	Set1, MLL, Set7/9, SMYD3, JAR1D1A
H3K9me	Euchromatin	Gene silencing	G9a; Suv91, StB1, PRD14, CLL8, GLP, Suv39h1, Suv39h2
H3K9me2	Euchromatin	Gene silencing	G9a; Suv91, StB1, PRD14, CLL8, GLP, Suv39h1, Suv39h2, JMJD2A
H3K9me3	Promoters and heterochromatin, Gene coding region	Gene silencing Gene activation	G9a; Suv91, StB1, PRD14, CLL8, GLP, Suv39h1, Suv39h2, JMJD2A
H3K27me1	Heterochromatin	Gene activation	
H3K27me2/3	Inactive-X chromosome, homeotic genes	Gene silencing	EZH2
H3K36me	Promoter	Not well characterized	JHDM1A
H3K36me2	Near double strand breaks, for repair	Gene silencing	NSD1, JMJD2A, JHDM1A
H3K36me3	3′ End of active genes. Marks exons.	Gene activation	JMJD2A
H3K79me2		Gene activation	Dot1L
H3K79me3		Gene activation	Dot1L
H4K20me1	Cell cycle regulation, present at active promoters	Gene activation	SET8/PR-Set7
H4K20me2	Heterochromatin, marks origin for replication, DNA damage response	Gene silencing	NSD1, Suv4-20h1, Suv4-20H2, Set8/PR-SET7
H4K20me3	Heterochromatin, at promoters	Gene silencing	NSD1, Suv4-20h1, Suv4-20H2, Set8/PR-SET7
